# Harnessing Microbiome-Mediated and Macrophage-Driven Mechanisms for Oral Wound Healing

**DOI:** 10.3390/microorganisms14020330

**Published:** 2026-01-30

**Authors:** Keerthi Priya Chinniampalayam Sekar, Bianca Schmiliver, Paige Elizabeth Pieterick, Tim Cha, Helly A. Patel, Hope Robinson, Prashant Kumar, David T. Wu, Rheinallt Jones, Steven Goudy

**Affiliations:** 1 Department of Otolaryngology, Emory University School of Medicine, Atlanta, GA 30322, USA; keerthi.priya.chinniampalayam.sekar@emory.edu (K.P.C.S.); biancaschmil@gmail.com (B.S.); tim.cha@emory.edu (T.C.); hpate58@emory.edu (H.A.P.); mhrobin@emory.edu (H.R.); 2 The Wallace H. Coulter Department of Biomedical Engineering, Georgia Tech and Emory, Atlanta, GA 30332, USA; ppieterick3@gatech.edu; 3 Department of Pediatrics, Center of CF and Airways Disease Research, Immunology and Molecular Pathogenesis, Emory University, Atlanta, GA 30322, USA; prashant.kumar@emory.edu; 4 Department of Oral Medicine, Infection, and Immunity, Harvard School of Dental Medicine, Boston, MA 02115, USA; davidwu.dmd@gmail.com; 5 Harvard John A. Paulson School of Engineering and Applied Sciences, Harvard University, Cambridge, MA 02139, USA; 6 Wyss Institute for Biologically Inspired Engineering, Harvard University, Boston, MA 02215, USA; 7 Division of Periodontics, Faculty of Dental Medicine and Oral Health Sciences, Montreal, QC H3A 1G1, Canada; 8 Division of Gastroenterology, Hepatology, and Nutrition, Department of Pediatrics, Emory University School of Medicine, Atlanta, GA 30322, USA; rjones5@emory.edu; 9 Children’s Healthcare of Atlanta 1405 Clifton Rd N E, Atlanta, GA 30322, USA

**Keywords:** oral wound healing, oral microbiome, macrophage polarization, inflammation resolution, host–microbe interactions, microbiome-based therapeutics

## Abstract

Oral mucosa healing is a complex process that involves the innate wound healing system, including the coagulation cascade, extracellular matrix remodeling, immune cell responses, and fibroblast and epithelial responses, within the context of a dynamic resident microbiome. Unlike cutaneous wounds, oral wounds heal rapidly with minimal scarring despite constant exposure to diverse microbial communities, saliva, and mechanical stress. Emerging evidence highlights the critical interplay between microbiome-mediated signaling and macrophage plasticity in shaping wound outcomes, suggesting that similar mechanisms operate within the oral cavity. Inflammation is an essential component of wound repair, and its resolution is necessary to promote tissue remodeling and functional regeneration. Macrophages play a central role in this transition through phenotype switching from a pro-inflammatory (M1) to a pro-resolving, anti-inflammatory (M2) state. This review synthesizes current understanding of the oral microbiome’s influence on macrophage polarization across distinct stages of oral wound healing and examines microbial-based strategies that modulate the immune response to enhance repair. Significant knowledge gaps remain, including limited clinical translation, inter-individual variability in microbiome composition, and complete mechanistic insight into host–microbe immune interaction. Addressing these challenges enables the development of precision microbiome-based therapeutics that restore microbial balance, direct macrophage-driven regeneration, and improve outcomes in oral wounds and chronic inflammatory conditions.

## 1. Introduction

Wound healing is a coordinated physiological process involving four overlapping phases—hemostasis, inflammation, proliferation, and remodeling—regulated through interactions among extracellular matrix components, immune cells, cytokines, chemokines, and growth factors [1]. Wounds are typically classified into acute and chronic. Acute wounds heal within 14 days and mostly occur as a result of burns, surgical sites and trauma, and are affected by factors including moisture, temperature and nutrients [2]. Chronic wounds are those that are delayed or fail to heal on their own within 14 days, and occur in conditions like venous ulcers, pressure ulcers, and diabetic ulcers, typically due to unresolved inflammation or impaired tissue regeneration [3]. Beyond host factors, the microbiome is now recognized as a critical regulator of the wound microenvironment. Eubiosis, defined as a balanced microbial community, is associated with efficient healing, whereas dysbiosis promotes sustained inflammation and chronic wound formation [4].

This microbial influence is particularly relevant in the oral cavity, where constant exposure to saliva, mechanical forces from mastication, and a highly diverse microbiota creates a uniquely dynamic healing environment [5]. The oral microbiome, the second largest microbial ecosystem after the gut, comprises approximately 700 bacterial species [6]. In health, commensal organisms maintain immune homeostasis, whereas dysbiosis characterized by the overrepresentation of pathobionts such as Streptococcus mutans, Porphyromonas gingivalis, Enterococcus faecalis, and Fusobacterium nucleatum is associated with inflammation, tissue breakdown, and impaired wound healing, as exemplified in periodontitis [7,8]. Immune regulation is central to this host–microbe interplay. Following injury, innate immune cells are recruited to the wound in response to cytokines and chemokines released during the hemostatic and inflammatory phases [9]. Macrophages orchestrate the transition from inflammation to tissue repair through a shift from a pro-inflammatory (M1) to a pro-regenerative (M2) phenotype [10]. Emerging evidence indicates that the oral microbiome is a key modulator of this phenotypic transition: eubiotic communities promote timely resolution of inflammation, enhance angiogenesis, and reduce scarring, whereas dysbiosis sustains M1 polarization and prevents progression into the regenerative phase [11,12].

Despite these insights, therapeutic strategies specifically targeting oral wound healing remain limited, and post-surgical complications related to microbial imbalance are common [13]. While cutaneous wound care has begun to incorporate microbiome-based approaches, including probiotic therapies and microbial metabolite delivery to modulate immune responses and enhance macrophage-driven repair, similar strategies for oral wounds are underdeveloped [14,15]. This review uniquely integrates current understanding of oral mucosal structure, immune regulation, and microbiome–macrophage interactions to define how microbial communities shape each phase of oral wound healing and to highlight emerging microbiome-based therapeutic strategies aimed at promoting regenerative repair.

## 2. The Oral Microenvironment: Structure, Immunity and Microbial Exposure

Antony van Leeuwenhoek, often hailed as the father of microbiology, was the first to visualize and identify oral bacteria [16]. The term oral microbiome collectively denotes the microorganisms inhabiting the human oral cavity. The oral microbiome, encompassing the collective of microorganisms within the oral cavity, constitutes the second largest microbial community in humans, following the gut microbiome. Using a microscope of his own construction, he made groundbreaking observations, discovering both protists and bacteria. In a noteworthy instance in 1674, he experimented with his own dental plaque and documented the presence of “little living animalcules which are prettily moving” [17]. The oral microbiome is a dynamic ecosystem where the core microbiome is present in all individuals, while the variable microbiome is affected by lifestyle and physiological differences, leading to a wide range of protein functions [18]. Within the oral cavity, microorganisms find distinct niches on both hard and soft surfaces which change over time, such as teeth, tongue, cheeks, gingival sulcus, tonsils, and palate [19]. These surfaces, coated with a complex bacterial biofilm, provide a rich environment for microbes to flourish. The oral cavity, along with the associated nasopharyngeal regions, offers an ideal environment for microbial growth. With a stable average temperature of 37 °C, and a consistent pH of 6.5–7 in saliva, which are favorable conditions for many bacterial species, the oral environment supports microbial survival, hydration, and nutrient transportation. In this intricate ecosystem, the oral microbiome plays a crucial role, contributing to both oral and systemic health [20].

The oral microbiome is made up of a core group of microorganisms that are shaped by factors such as diet, hygiene, immunity, medications, and overall health, which are common among individuals but can also vary. [20]. Disruption in the microbial balance that leads to diseases is termed dysbiosis. Pathogens such as Porphyromonas ginigivalis, Fusobacterium nucleatum and Candida albicans have been implicated in periodontitis, endodontic infections and failed root canal treatments [21].

Beyond bacteria, the microbiome includes a viral community (the virome) that actively shapes host immunity. Predominantly composed of bacteriophages, the virome regulates microbial ecology through lytic and lysogenic cycles, horizontal gene transfer, and modulation of bacterial virulence and biofilm dynamics. Virome dysbiosis can directly influence inflammatory signaling and immune activation, indicating that dysbiosis-driven inflammation cannot be fully explained by bacterial communities alone. This perspective expands the mechanistic framework beyond bacteriocentric models [22].

Viruses like human cytomegalovirus [23], herpes simplex virus [24], Epstein–Barr virus [25], human papilloma virus and human herpesvirus [26] are associated with oral pathology. Structurally the oral mucosa comprises stratified squamous epithelium, basal lamina, lamina propria and sometimes submucosa [27]. It has a unique environment unlike the skin, both anatomically and immunologically, that lacks appendages such as hair follicles and sebaceous glands, limiting stem cell reservoirs [28]. The gingiva and hard palate are keratinized due to mechanical stress, whereas other regions remain non-keratinized [29].

Skin-resident immune cells, comprising both myeloid and lymphoid lineages, are essential for maintaining tissue homeostasis and orchestrating immune responses [22]. Langerhans cells in the epidermis and dermal dendritic cells within the papillary dermis serve as pivotal antigen-presenting cells [23]. Macrophages and monocyte-derived macrophages are part of the innate immune system in the dermis that mediate phagocytosis and secrete cytokines and growth factors critical for tissue repair and remodeling [24]. Mast cells and eosinophils contribute to allergic inflammation and parasitic defense, whereas neutrophils rapidly infiltrate sites of infection or injury to eliminate pathogens and facilitate angiogenesis [25]. Humoral immunity, including T lymphocyte subsets such as CD4^+^, CD8^+^, and γδ T cells, modulates cutaneous immune responses and promotes wound healing [26]. B cells primarily mediate antibody production and regulate immune homeostasis within the skin microenvironment [27]. Differences in reduced neutrophil and macrophage infiltration in oral wounds, compared to cutaneous wounds, are associated with reduced expression of pro-fibrotic cytokines like TGF-1, which, in part, explains the lack of scarring after oral injury [24]. Immune homeostasis and tissue integrity within the oral cavity are maintained by commensal organisms like Streptococcus salivarius and Actinomyces naeslundii; however, pathogenic microbes may disrupt this balance through the secretion of virulence factors like lipopolysaccharide (LPS), outer membrane vesicles (OMV) and peptidoglycans (PG) that influence macrophage polarization [28].

Macrophages are differentiated from monocytes during the inflammatory phase of wound healing based on the signals from antigen-presenting cells and cytokines such as IL-1β and TNF-α produced from neutrophils and tissue-resident macrophages [30]. Macrophages can polarize into pro-inflammatory M1 or anti-inflammatory M2 phenotypes based on the environmental stimuli and will secrete pro-inflammatory or pro-regenerative cytokines, respectively [31]. The oral microbiome can modulate the polarization of macrophages to an inflammatory M1 phenotype in a dysbiotic environment and a pro-regenerative M2 phenotype in a eubiotic state, thereby impacting the wound outcomes, either by promoting resolution of inflammation or contributing to chronic inflammation [32].

## 3. The Impact of Oral Microbiome on Wound Healing Phases

**Figure 1:**
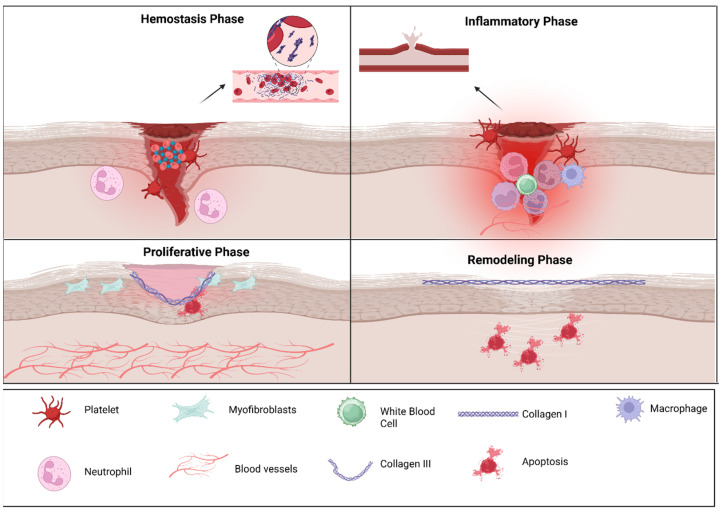
Cellular players of oral wound healing: The figure illustrates the four principal phases of wound repair. During hemostasis, platelet aggregation and clot formation occur to rapidly control bleeding. In the inflammatory phase, infiltrating neutrophils and macrophages clear debris and pathogens while releasing cytokines that guide subsequent repair. The proliferative phase is characterized by myofibroblast activity, extracellular matrix deposition, angiogenesis, and re-epithelialization to restore tissue continuity. In the remodeling phase, collagen III is gradually replaced by collagen I, and extracellular matrix reorganization enhances tensile strength as excess cells undergo apoptosis. Key cellular and structural components involved in each phase are indicated in the legend. The above figure was created with BioRender.com. Goudy, S. (2026) https://BioRender.com/hgymzt8.

Healing of the oral mucosa occurs in four distinct stages—hemostasis, inflammation, proliferation and remodeling—driven by coordinated actions of immune cells, particularly macrophages, which help in the transition between pro-inflammatory and pro-repair phenotypes, and the molecular signals shaped by the microbiome (Figure 1) [33].

### 3.1. Hemostatic Phase

Immediately following tissue injury, vascular disruption initiates the hemostatic clotting cascade to prevent blood loss and create a matrix for cellular infiltration. Platelet activation leads to fibrin clot formation and release of vasoactive agents, cytokines such as IL-1β and TNF-α, and growth factors such as PDGF and TGFβ that recruit immune cells and contribute to ECM remodeling (Figure 1, Table 1) [5]. The release of these cytokines from platelets following wounding incites a local and systemic inflammatory response that induces inflammatory cell migration of neutrophils and macrophages into the wound bed. The fibrin clot acts as a scaffold for the neutrophils and macrophages to migrate into the wound and initiate wound repair. The fibrin matrix is rapidly stabilized by interaction with salivary components like proline-rich proteins, statherin, and histatins that facilitate cell adhesion and early epithelial migration [34]. Saliva also contributes antimicrobial peptides like defensins, cathelicidins, and histatins and enzymes that control bacterial colonization [35]. Ultimately the release of anti-inflammatory mediators such as lipoxins, resolvins, and protectins helps resolve inflammation and prevent excessive clotting and tissue damage [36]. Saliva contains tissue factor and histatins that quickly promote platelet aggregation and the formation of fibrin, operating faster than the coagulation process in the skin. Chewing increases saliva production to help clear away debris while the biofilm helps secure the clot against early microbial attacks [1].

**Table 1:** Pro-inflammatory and anti-inflammatory mediators in oral wound healing.

Wound Healing Phase	Description	Role of Inflammatory Markers	Role of Anti-Inflammatory Markers	References
**Hemostasis phase**	Initial response to injury involving blood clot formation.	Platelets release cytokines (e.g., IL-1β, TNF-α) and growth factors (e.g., PDGF, TGF-β) to initiate inflammation and recruit immune cells to the wound site.	Anti-inflammatory mediators such as lipoxins, resolvins, and protectins help resolve inflammation and prevent excessive clotting and tissue damage.	[37,38,39]
**Inflammatory** **phase**	Characterized by inflammation and removal of debris.	Neutrophils and macrophages release pro-inflammatory cytokines (e.g., IL-6, IL-8, TNF-α) to eliminate pathogens and cellular debris and stimulate angiogenesis and fibroblast proliferation.	Interleukin-10 (IL-10) and transforming growth factor-beta (TGF-β) help suppress inflammation, regulate immune responses, and promote tissue repair.	[40,41,42]
**Proliferative phase**	Formation of new tissue by fibroblasts and endothelial cells.	Fibroblasts produce collagen and extracellular matrix components, while endothelial cells promote angiogenesis and blood vessel formation.	Anti-inflammatory cytokines like IL-4 and IL-13 help modulate the inflammatory response and facilitate tissue remodeling and wound closure.	[43,44,45]
**Remodeling phase**	Maturation and remodeling of the newly formed tissue.	Matrix metalloproteinases (MMPs) produced by fibroblasts and macrophages degrade excess collagen and promote tissue remodeling.	Tissue inhibitors of metalloproteinases (TIMPs) control MMP activity, prevent excessive tissue degradation, and promote tissue maturation and strength.	[46,47,48]

### 3.2. Inflammatory Phase

The inflammatory phase begins within hours post-injury in response to the cytokines released during the hemostatic phase (IL-1β, TNF-α, etc.) and peaks between 24 and 72 h. Neutrophils are the first response, tasked with clearing microbial and cellular debris through the release of reactive oxygen species (ROS), proteolytic enzymes, and neutrophil extracellular traps (NET) [49]. Neutrophils produce CCL2, a chemokine responsible for monocyte recruitment to inflamed tissue [50], and macrophages are also indirectly attracted by cytokines such as IL-1β and TNF-α [51]. GM-CSF [52] also plays a role to attract systemic monocytes, which gradually replace the neutrophils and differentiate into macrophages at the wound site [53]. The impact of the microbiome on neutrophil response is that eubiosis supports a balanced neutrophil response while dysbiosis can lead to an overactive or dysfunctional neutrophil response, hindering healing and prolonging the inflammatory phase [54].

Macrophages are the principal orchestrators of the inflammatory phase of wound healing, initially polarizing monocytes towards a classically activated M1 phenotype in response to pathogen-associated molecular patterns (PAMPs) that are found in bacteria (LPS) and by pro-inflammatory cytokines like IFN-γ and TNF-α. M1 macrophages release IL-1β, IL-6, TNF-α and inducible nitric oxide (iNOS) [55]. The secretion of these pro-inflammatory cytokines is crucial for microbial clearance and activation of downstream immune responses. Pro-inflammatory cytokines can also enhance vascular permeability and recruit neutrophils, monocytes and Th17 cells. The acute phase response of inflammatory wound healing is promoted by B cell differentiation and by the production of IL-6. Persistent inflammation leads to the excessive production of inflammatory cytokines, increasing the production of ROS, which leads to tissue damage and is perpetuated by the continuous recruitment and activation of neutrophils and M1 macrophage cells. Moreover, chronic stimulation of fibroblasts and endothelial cells under the influence of M1-derived cytokines results in the formation of persistent granulation tissue by neovascularization and collagen deposition, which lacks the maturation of reparative tissue. The inhibition of the shift to reparative M2 from M1 impairs wound healing, resulting in fibrosis and the formation of a chronic, non-healing wound [56]. The lack of interleukin-10 (IL-10) and transforming growth factor-beta (TGF-β) secretion by M2 macrophages allows unchecked inflammation, an ongoing immune response, and failure of tissue repair.

The beneficial microbes, such as Streptococcus salivarius and Actinomyces naeslundii, in a state of eubiosis help suppress excessive inflammation. The activity of these two bacterial strains during oral wound healing enhances epithelial barrier integrity and stimulates signaling pathways that favor M2 polarization. Together, these bacterial strains contribute to the resolution of the inflammatory phase of wound healing and begin the proliferative phase with rapid wound closure and minimal fibrosis [57]. However, dysbiosis marked by pathogenic species such as Porphyromonas gingivalis and Fusobacterium nucleatum sustains M1 activation through persistent stimulation by virulence factors including LPS and outer membrane vesicles [58]. The presence of LPS and OMV in the wound healing environment engages toll-like receptors (TLRs) on immune cells, which prevents transition to the M2 macrophage phenotype and prolongs the M1 macrophage phenotype, leading to unresolved inflammation and contributing to impaired healing or chronic wounds [59]. The persistent M1 phase of macrophages, associated with dysbiosis and inflammatory response to LPS and OMVs, leads to chronic inflammation, which is demonstrated by the continued production of inflammatory cytokines IFN-γ, TNF-α, IL-1β, IL-6, TNF-α and inducible nitric oxide (iNOS), all of which contribute to persistent inflammation [60].

The state of oral eubiosis, characterized by the presence of beneficial microbes such as Streptococcus salivarius and Actinomyces naeslundii, plays a crucial role in resolving inflammation and promoting tissue repair [57,61]. These commensals help suppress excessive immune responses, enhance epithelial barrier integrity, and activate signaling pathways that favor M2 macrophage polarization. This shift from the pro-inflammatory M1 to the anti-inflammatory M2 phenotype facilitates the resolution of the inflammatory phase and the initiation of the proliferative phase of wound healing, resulting in rapid wound closure with minimal fibrosis [57]. Additionally, microbial metabolites such as short-chain fatty acids (SCFAs) and polysaccharide A from Bacteroides fragilis contribute to this effect by dampening TLR signaling, reducing NF-κB activation, and promoting regulatory immune responses conducive to M2 polarization [62].

In contrast, dysbiosis, marked by an overrepresentation of pathogenic species like Porphyromonas gingivalis, Fusobacterium nucleatum, and Escherichia coli, sustains M1 macrophage activation through persistent exposure to virulence factors such as LPS and OMV [58]. These microbial products stimulate toll-like receptors (especially TLR4) on immune cells, reinforcing NF-κB and STAT1 signaling [63]. This prolongs the M1 phase, characterized by the continued release of pro-inflammatory cytokines like IL-1β, TNF-α, and inducible nitric oxide synthase (iNOS)-derived nitric oxide [59]. As a result, the inflammatory phase is abnormally extended, impairing the transition to the M2 phenotype and leading to chronic inflammation, excessive tissue damage, and a failure to progress to the proliferative phase. This sustained M1 activity disrupts granulation tissue remodeling, promoting fibrosis and the development of chronic, non-healing wounds [64]. Salivary antimicrobial agents such as defensins and IL-10 cytokines rapidly manage neutrophil infiltration, in contrast to the prolonged pro-inflammatory period seen in skin. The act of chewing physically primes the immune system for prompt elimination, as biofilm encourages the modulation of commensals rather than fostering dysbiosis [2].

### 3.3. Proliferative Phase

The proliferative phase of wound healing is indicated by formation of granulation tissue, re-epithelialization, and neovascularization [1]. A key component of the proliferative phase of wound healing occurs post-injury, under the influence of IL-4, IL-10 and IL-13, by alternatively activated M2 phenotype macrophages [65]. Anti-inflammatory cytokines and growth factors produced by M2 macrophages, including TGF-β, VEGF- and IGF-1, promote angiogenesis, fibroblast activation and ECM deposition [66]. During the proliferative phase of wound healing, fibroblasts infiltrate the wound bed and initiate granulation tissue formation through the secretion of type 1 collagen and fibronectin [67]. Endothelial cells form new capillaries, restoring tissue perfusion and supporting epithelial regeneration accelerated by salivary histatins and growth factors [13]. Anti-inflammatory cytokines like IL-4 and IL-13 help modulate the inflammatory response and facilitate tissue remodeling and wound closure [66]. EGF and salivary growth factors promote the rapid migration of keratinocytes and the formation of new blood vessels, allowing oral wounds to heal in days compared to the weeks required for skin wounds. Regular chewing stimulates fibroblast mechanotransduction, helping to control contraction, while biofilm signaling enhances the optimal deposition of the extracellular matrix [2].

The oral environment is colonized by a complex and diverse microbiota that can significantly impact wound healing dynamics along with a constant presence of saliva that contains antimicrobial peptides (defensins, histatins, etc.) [68], enzymes and growth factors (EGF), vascular endothelial growth factor (VEGF), transforming growth factor alpha (TFG-α), transforming growth factor beta (TFG-β), nerve growth factor (NGF), fibroblast growth factor (FGF), and insulin-like growth factor (IGF) [69], which facilitate re-epithelization and angiogenesis, modulating the wound healing dynamics. The proliferative phase is disrupted by dysbiosis, prolonged inflammation, impaired fibroblast function and degrading ECM components [70]. Dysbiosis also modulates macrophage polarization, promoting pro-inflammatory M1 phenotypes, thereby delaying progression to granulation tissue formation and re-epithelization [12].

### 3.4. Remodeling Phase

Remodeling of oral wounds begins as early as one-week post-injury in soft tissue (e.g., oral mucosal wound) and may extend over several weeks or months in hard tissue (e.g., alveolar bone followed dental extraction) depending on the wound type and size. During this remodeling phase, fibroblasts differentiate into myofibroblasts, primarily driven by transforming growth factor (TGF-β1) signaling through the SMAD pathway and supported by platelet-derived growth factor (PGDF) and cytokines like interlukein-6 (IL-6), enabling wound contraction. Matrix metalloproteinases (MMP) produced by macrophages and fibroblasts degrade excess ECM proteins, which allows tissue architecture to be restored and prevents excessive fibrosis [71,72,73]. Meanwhile the tissue inhibitors of metalloproteinase (TIMPs) prevent excessive degradation of the ECM to maintain tissue homeostasis [74]. Pro-resolving features of a eubiotic oral microbiome reduce inflammation and fibrosis and promote the restoration of mucosal architecture by promoting the activity of M2 macrophages [75]. Saliva balances MMPs and TGF-β to create structured collagen alignment without fibrosis, unlike scars in the skin. Masticatory pressure enhances tensile strength adaptively, while consistent biofilms maintain vascular stability [2].

In oral tissues, remodeling is typically faster and results in less scarring than in cutaneous skin due to multiple factors including a lower fibrotic response and reduced production of TGF-β1 [76]. However, in the presence of dysbiosis and chronic inflammation, the proliferative phase may be disrupted by sustained pro-inflammatory signals, impaired macrophage polarization, and dysregulated TGF-β1 [77]. The repeated tissue injury from chronic inflammation and impaired barrier function leads to delayed healing, chronic inflammation or tissue breakdown, as observed in periodontitis and oral mucosal ulcers, and ultimately a fibrotic unhealed oral wound [78].

The following table summarizes the four major phases of wound healing hemostasis, inflammation, proliferation, and remodeling. It highlights the biological processes occurring in each phase and the contributions of key inflammatory and anti-inflammatory mediators involved in regulating tissue repair and restoration.

## 4. Eubiosis and Dysbiosis vs. Probiotics in Skin, Gut and Oral Habitat

**Figure 2:**
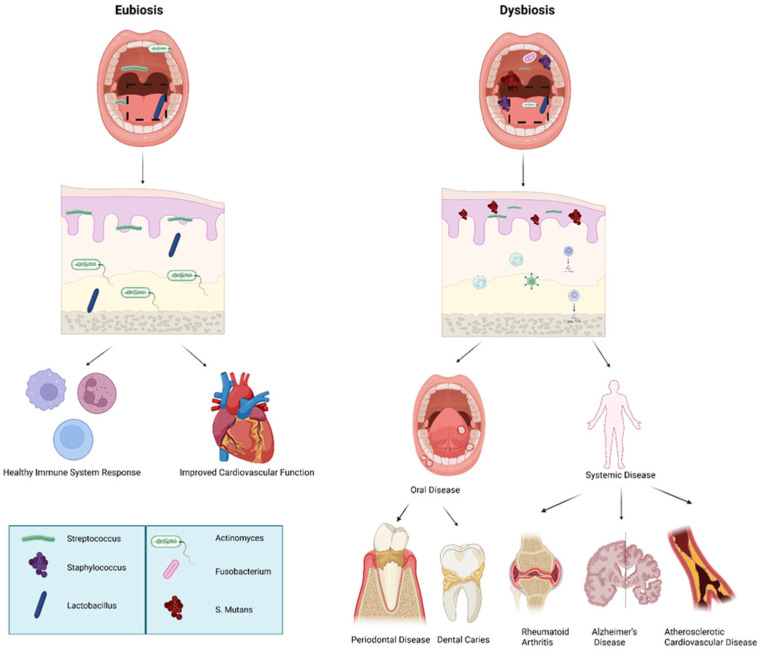
Eubiotic vs. dysbiotic microbiome in human health and oral disease. Overview of oral microbiome balance (eubiosis) versus imbalance (dysbiosis) and associated systemic outcomes: Eubiosis is characterized by a predominance of beneficial commensal bacteria such as *Streptococcus*, *Staphylococcus*, and *Lactobacillus*, supporting mucosal integrity, balanced immune signaling, and improved cardiovascular health. In contrast, dysbiosis features increased pathogenic microorganisms including *Fusobacterium*, *Actinomyces*, and *Streptococcus mutans*, leading to oral disease (periodontal disease, dental caries) and contributing to systemic conditions such as rheumatoid arthritis, Alzheimer’s disease, and atherosclerotic cardiovascular disease. The above figure was created with BioRender.com. Goudy, S. (2026) https://BioRender.com/1o6wsje.

Eubiosis is the condition in which the host and its microbiota maintain a healthy equilibrium, characterized by microbial diversity and interactions that promote tissue homeostasis and immune regulation [79]. A dysregulation or misbalance in the host’s healthy microbiota is termed as dysbiosis, which often occurs in a range of conditions beginning in the oral cavity, including oral diseases such as periodontitis and dental caries; gastrointestinal disorders such as Clostridium difficile colitis, antibiotic-associated diarrhea, irritable bowel syndrome (IBS), and inflammatory bowel diseases (e.g., Crohn’s disease and ulcerative colitis); and systemic diseases including metabolic disorders (e.g., obesity and type 2 diabetes), allergies, and atopic dermatitis [80]. Probiotics are the beneficial bacteria that are used to maintain and/or restore eubiosis (Figure 2, Table 2) [81].

**Table 2:** Comparison of microbiota in distinct ecology in eubiosis and dysbiosis: Comparison of microbiota composition under eubiosis versus dysbiosis across major body sites. Representative probiotic or beneficial taxa associated with eubiosis are listed alongside pathogenic or dysbiosis-associated microorganisms. Reference numbers correspond to supporting studies in the text.

Conditions	Eubiosis vs. Probiotic	Dysbiosis vs. Pathogenic
Gut/intestinal microbiota	*Bacteroides fragilis* [82] *Faecalibacterium prausnitzii* [83] *Bifidobacterium breve* [84] *Lactobacillus rhamnosus GG* [85], *Bifidobacterium longum* [86,87]	*Clostridium difficile* [88], *Escherichia coli* [89], *Enterococcus faecalis* [90]
Oral microbiota	*Streptococcus salivarius* [91], *Actinomyces naeslundii, Streptococcus salivarius K12* [92], *Lactobacillus reuteri* [93]	*Streptococcus mutans* [94], *Porphyromonas gingivalis* [95], *Fusobacterium nucleatum* [96]
Skin microbiota	*Staphylococcus epidermidis* [97], *Cutibacterium acnes* [98], *Corynebacterium accolens* [99], *Lactobacillus plantarum* [100], *Lactobacillus rhamnosus GG* [101]	*Staphylococcus aureus* [102], *Malassezia* spp. [103]
Vaginal microbiota	*Lactobacillus crispatus* [104], *Lactobacillus iners* [105], *Lactobacillus gasseri* [106], *Lactobacillus rhamnosus GR-1* [107], *Lactobacillus reuteri RC-14* [108]	*Gardnerella vaginalis (BV)* [109], *Candida albicans (yeast infection)* [110], *Atopobium* [110]

## 5. Macrophage Polarization in Oral Wound Healing

Macrophages are highly plastic innate immune cells capable of shifting their phenotypic and functional profiles in response to environmental cues. This dynamic transition, known as macrophage polarization, is guided by a variety of cytokines, surface markers and receptors [111]. In the context of oral wound healing, macrophages exhibit phase-specific roles—initially contributing to pathogen clearance and inflammation via the M1 phenotype and later facilitating tissue regeneration and resolution through the M2 phenotype [112].

### 5.1. M1 and M2 Phenotypes and Function

During the inflammatory phase of wound healing, macrophages adopt a classically activated M1 phenotype in response to microbial ligands (e.g., LPS, flagellin) and pro-inflammatory cytokines (e.g., IFN-γ) [113]. M1 macrophages are characterized by CD86, MHC class II, CCR7, CX3CR1 and inducible nitric oxide synthase (iNOS) [114]. M1 macrophages secrete high levels of TNF-α, IL-β, IL-6, IL-12 and ROS, which collectively contribute to debris clearance and pathogen elimination. However, sustained M1 activation, especially in dysbiotic oral environments, can contribute to chronic inflammation and impaired healing [115].

As wound healing transitions into the proliferative phase, macrophages begin to polarize from M1 to M2 phenotypes. This is stimulated by cytokines such as IL-4, IL-10, IL-13 and TGF-β. M2 macrophages are marked by expression of CD68, CD163, CD206, PD-L1 and PD-L2, with constitutive CSFIR expression playing a supportive role [116]. These cells promote an anti-inflammatory microenvironment by secreting cytokines and growth factors like IL-10, TGF-β, VEGF and IGF-1, which drives fibroblast activation, angiogenesis, extracellular matrix deposition and tissue regeneration [117].

In the remodeling phase, the high expression of CD206 and CD163 supports proper tissue structure restoration. PD-L1 and PD-L2 further help maintain tissue homeostasis [118]. M2 macrophages thus play an essential role in scar resolution and epithelial regeneration [119].

Importantly, macrophage polarization exists on a functional spectrum rather than as a binary switch. Mixed M1/M2 profiles are commonly observed in chronic oral wounds and biofilm-associated infections, illustrating the complexity of immune–microbe interactions in the oral microenvironment [120].

Oral wound healing involves a finely tuned immunological response orchestrated by macrophage plasticity and phenotypic shifts in response to dynamic cues such as cytokines, microbial factors and mechanical stress seen in oral wound healing [121]. Disruption in this balance, particularly prolonged M1 activation or insufficient M2 transition, can lead to chronic inflammation and delayed wound healing [64].

### 5.2. Microbial Influence on Macrophage Polarization

**Figure 3:**
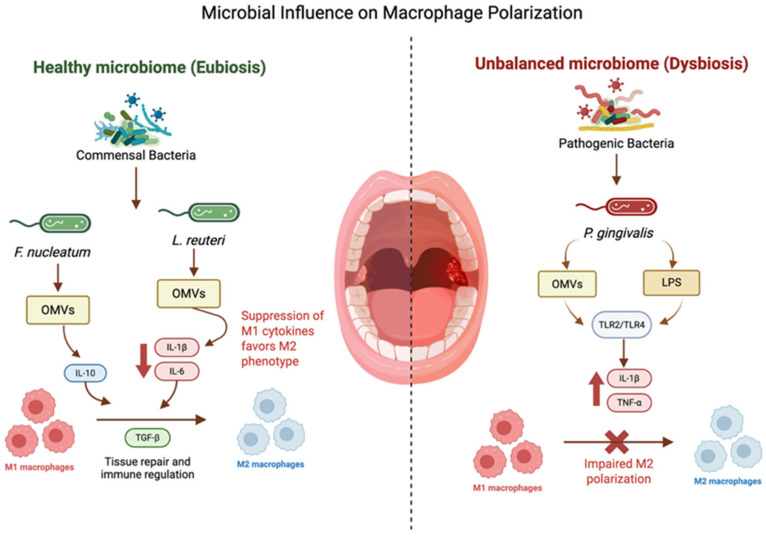
Microbial influence on macrophage polarization: bacterial OMVs modulate macrophage polarization: *F. nucleatum* promotes M2-like traits, *P. gingivalis* induces inflammatory M1 responses and pyroptosis, and *L. reuteri* shifts macrophages toward M2 and enhances phagocytosis. The above figure was created with BioRender.com. Goudy, S. (2026) https://BioRender.com/ulcvvbs.

Microbial products are key regulators of macrophage function in oral tissues. Commensal and probiotic bacteria can promote M2 polarization and wound resolution, whereas pathogenic species often sustain or exacerbate M1-driven inflammation. Examples of the bacterial impact on macrophage phenotypes are well described [122] (Figure 3). These interactions suggest that targeted modulation of the microbiome may shape macrophage responses and redirect wound trajectories towards resolution rather than chronicity Table 5.

## 6. Microbiome-Based Therapeutics for Modulation of Wound Healing

The microbiome plays a dual role in oral wound healing, capable of supporting immune homeostasis or promoting chronic inflammation depending on its composition [123]. Increasing evidence suggests that therapeutic modulation of microbiomes, particularly through the delivery of probiotics or microbial metabolites, can influence macrophage polarization and improve wound healing outcomes [4]. Insights from immune-targeted microbiome therapies in chronic inflammatory diseases further support the feasibility of translating these approaches to wound healing contexts, where similar immunoregulatory mechanisms are operative [124]. Here, we review preclinical and clinical strategies that leverage microbial interventions across oral and systemic wound models.

Although preclinical results are encouraging, there are notable challenges in translating microbiome therapeutics into clinical practice. The stability of strains is threatened by factors such as salivary flow, fluctuations in pH, and mechanical disruption caused by chewing, which affect the viability of probiotics. The ability of these external strains to persist in colonization is generally low, as they face strong competition from established oral biofilms. There are safety issues to consider, including the risk of opportunistic infections and the transfer of antibiotic resistance genes in susceptible patients, while regulatory hurdles require thorough strain characterization and clinically relevant endpoints that are not yet established for applications related to oral wounds [56].

### 6.1. Probiotic-Based Therapeutics in Oral and Cutaneous Wounds

Probiotics like *Lactobacillus reuteri*, *Bifidobacterium breve* and *Streptococcus salivarius* have been explored in various wound contexts, with promising immunomodulatory effects [124]. These bacteria modulate local inflammation, influence cytokine profiles and promote a shift towards reparative M2 macrophage phenotypes [64] Table 4.

An advanced delivery system is needed to improve localization, preserve viability and ensure controlled therapeutic activity in vivo, particularly for mammalian cells, genetically engineered microbes, viruses, etc. [125]. Depending on the target tissue and desired therapeutic outcome, these agents can be administered through oral, topical or local application, intravenous and intratumoral injection, and surgical implantation using biomaterial-based scaffolds; among other delivery platforms, hydrogels typically composed of natural polymers such as alginate, collagen and chitosan or synthetic polymers such as polyethylene glycol (PEG) are the most extensively explored carriers. These carriers enable encapsulation, prolonged local retention, protection from antagonistic environments, and sustained release of living therapeutics [126]. In addition, direct delivery of living cells and bacteria including stem cells, CAR-T cells, dendritic cells and engineered microbes into target tissues with enhanced spatial control and retention is performed by minimally invasive biomaterial-based microneedles [127]. Supplemental approaches such as conjugation of aptamers, antibodies and other affinity ligands to the surface of bacteria and mammalian cells have been developed to improve precision and minimize off-target effects [128].

### 6.2. Mechanisms of Microbial Modulation: Cytokine Signaling and Receptor Engagement

Beneficial bacteria modulate macrophage function through a variety of secretory products:

- Outer membrane vesicles (OMVs): OMVs from *F. nucleatum* and *P. gingivalis* influence TLR2/TLR4 signaling and activation of inflammation, with downstream effects on IL-1β, IL-6, TNF-α, and IFN-β production [129].
- Lipoteichoic acids and peptidoglycans: structural components of Gram-positive bacteria such as *S. mutans* and *A. naeslundii* stimulate release of pro-inflammatory cytokines like TNF-α, IL-1β, and IL-6 in macrophages [130].
- Lantibiotics (e.g., *salivaricins*): produced by *S. salivarius* K12, these peptides inhibit NF-κB activation and reduce IL-8 and IL-6 signaling, promoting a homeostatic environment [131].
- Metabolites (e.g., reuterin): produced by *L. reuteri*, reuterin exhibits anti-inflammatory properties by neutralizing LPS and suppressing macrophage polarization toward the M1 state [132].

### 6.3. Probiotic-Mediated M2 Polarization—A Therapeutic Opportunity

In vivo and in vitro, studies consistently demonstrate that select probiotic strains enhance the M2 polarization of macrophages, marked by increased CD163/CD206 expression and IL-10/VEGF/TGF-β production [75]. These immunological shifts are accompanied by improved re-epithelization, enhanced vascularization, reduced inflammatory infiltrate, and suppressed M1-associated cytokines [12]. These findings have important implications for translational wound care, particularly in managing oral wounds with chronic inflammation or microbial dysbiosis, including diabetic oral ulcers, periodontitis and postsurgical effects [13].

The active process whereby macrophages change the phenotype guided by various cytokines, cell surface markers and receptors in a plastic manner is referred to as polarization. M1 macrophages are characterized by the expression of CD86 [133], MHC class II [133], CCR7 [134], and CX3CR1 [135]. In the initial inflammatory phase, markers like TNF-α, IL-1β, IL-6, and IL-12 [136] signal to M1 macrophages, which are involved in the process of clearing debris and pathogens. In the proliferative phase, markers like PD-L1 [137], PD-L2 [138], and constant CSFIR expression promote an anti-inflammatory and repairing mechanism with M2 macrophage polarization.

Reduced inflammation and enhanced fibroblast activity are involved in extracellular matrix deposition and promote angiogenesis, facilitated by M2 macrophages that are characterized mainly by surface markers such as CD68 [139], CD163 [140], and CD206 [141]. Cytokines like IL-10 and growth factors like TGF-β and VEGF are produced by M2 macrophages and are involved in tissue regeneration, angiogenesis, and fibroblast activation in the proliferative stage [142]. High expression of CD206 and CD163 for ensuring proper tissue structure (especially in the remodeling phase [143] and additionally PD-L1 and PD-L2 are involved in homeostasis during this phase [144].

Oral wound healing is orchestrated by specific macrophage phenotypes and cytokines, and the phases involved are inflammatory, proliferative and remodeling. Prolonged or delayed wound healing is termed as chronic phase or chronic inflammation phase [145,146] Table 3.

**Table 3:** Role of macrophages, cytokines, and phases of healing in various oral wound conditions.

Phenotype	Cytokines/ Chemokines/Growth Factor	Role in Oral Wound Healing	Phases	Oral Wound Conditions	References
M1	IL-1β, IL-6, TNF-α, IFN-γ, ROS	Initiate the inflammatory response, clear pathogens, and recruit immune cells to the injury site.	Inflammatory Phase	Acute Oral Wounds (e.g., dental extraction, minor trauma)	[117,147]
M2	IL-10, TGF-β, VEGF, IGF-1	Suppress inflammation, promote angiogenesis, and stimulate fibroblast activity and extracellular matrix deposition.	Proliferative Phase	Chronic Periodontal Wounds (e.g., periodontitis)	[148]
M2	M2b-like (Immunoregulatory) IL-10, IL-1RA, TGF-β	Modulate immune responses, balancing pro- and anti-inflammatory signals.	Transitional Phase	Diabetic Oral Wounds (delayed healing)	[149]
M2	TGF-β, IL-10, PDGF, VEGF	Facilitate tissue remodeling, collagen deposition, and scar formation.	Remodeling Phase	Oral Mucosal Wounds (e.g., ulcers, post-surgery)	[150]
M1	TNF-α, IL-1β, IL-6 (low IL-10)	Persistent inflammation, chronic wounds, and impaired healing.	Chronic Wound Phase	Chronic Periodontitis	[151]
Mixed M1/M2 Phenotypes	IL-6, IL-10, VEGF, TGF-β, IFN-γ	Adapt to saliva, oral microbiota, and mechanical stress for tissue-specific repair.	Oral Wound Healing	Oral Wounds in Immunocompromised Patients	[152]
M1-	IL-1β, TNF-α, IL-6, IL-17	Delayed resolution of inflammation, impairing wound healing.	Chronic Inflammation	Oral Wounds in Smoking (e.g., impaired healing)	[153]
Mixed M1/M2	IL-1β, TNF-α, IL-6, IL-10	Biofilm-associated wounds trigger prolonged inflammation and microbial persistence.	Inflammatory/Chronic	Dental Implants and Peri-implantitis	[154]
Tumor-associated Macrophages (TAMs)	TGF-β, IL-10, VEGF, IL-6	Promote tumor invasion and immunosuppression but can support tissue healing in surgical resection wounds.	Variable	Oral Squamous Cell Carcinoma (OSCC) Wounds	[155]

This table highlights the different types of macrophages (M1, M2) and their associated cytokines, specific functions, and roles across different phases of wound healing in various oral conditions, including acute wounds, chronic periodontal wounds, diabetic oral wounds, and biofilm-related infections.

**Table 4:** Microbiome-based interventions in wound healing: use of microbiome interventions for wound healing, detailing their application methods, wound models, impact on macrophages, and overall effects on healing outcomes. N/A—Not applicable.

Source	Microbiome Intervention	Application	Wound Healing Model	Impact on Macrophages	Effect
Wälivaara D.Å 2019 (Clinical Trial) [156]	*Lactobacillus reuteri*-containing lozenges	Oral	Surgical extraction of impacted mandibular third molars	N/A	No significant influence of probiotics on wound healing mechanism. Did observe reduced swelling and pain
Li 2023 [157]	*Bifidobacterium breve*	Oral	C57BL/6N mice hard palate mucosal defect model	N/A	Therapeutic potential of *Bifidobacterium breve* on oral mucosal wound healing after impairment caused by Sunitinib. Promoted wound healing by intestinal dendritic cell-derived IL-10
Han 2020 [158]	*L. reuteri*	Inoculated in the wound	Palate mucosal model on C57BL/6 mice	N/A	Reuterin in *L. reuteri* neutralized the LPS in *L. gingivalis* and inhibiting inflammation while promoting wound healing
Xu 2024 [159]	*Lactobacillus paracasei* TYM202	Injection	Full-thickness skin injury on Sprague Dawley rats	Found higher levels of M2 macrophages	The probiotic hydrogel encapsulated with *L. paracasei TYM202* (HAEPS@L.sei gel) promotes wound healing by reducing inflammation and promoting angiogenesis and increasing collagen deposition
Li 2024 [160]	*Gluconacetobacter* (formerly *Acetobacter*), *Agrobacterium*, and *Aerobacter*		Skin wounds on the back of Sprague Dawley rats	N/A	Bacterial cellulose hydrogel (Ag-BCN) and carbon nanodots with resveratrol reduced wound healing time and ROS and promoted wound healing
Mohtashami 2021 [161]	*Lactobacillus bulgaricus* and *Lactobacillus plantarum*	Topical	Diabetic cutaneous wounds in Wistar rats	Promoted the proliferation of macrophages	The bacteria modulated the immune response and accelerated the wound healing process
Evelina Vågesjö 2018 [162]	*Lactobacilli* with a plasmid encoding CXCL12	Topical	Full-thickness wound on the hind limb of C57BL/6 mice	Promoted proliferation of TGF-β+ macrophages	Accelerated wound closure
Emelie Öhnstedt 2023 [163] (human clinical trial)	Engineered *Limosilactobacillus reuteri* R2LC to produce CXCL12-a (ILP100-Topical)	Topical	Full-thickness wound punching on the arm	N/A	Overall safe. Multiple doses resulted in larger amount of healed wounds

**Table 5:** Bacterial influence on macrophage phenotype and inflammatory responses: The effects of various bacterial species and their secretory products on macrophage phenotype and immune signaling. This includes details on cytokine modulation, inflammasome activation, and macrophage polarization (M1/M2).

Oral Microbiota Bacteria	Product/Secretory Factors	Expected Effects with Reference Links
*Streptococcus salivarius*	---	Inhibits NF-KB activation and IL-8 secretion (both use HT-29 primary tumor epithelial cells)
	---	Increases IL-6 and TNF-α expression (mentions “infected oral epithelial cells also expressed relatively high levels of the chemo attractive cytokine IL-8”)
*Actinomyces naeslundii*	Peptidoglycan purified	Increases IL-1β, IL-6, and TNF-α gene expression in mouse peritoneal macrophages (and stimulates osteoclast genesis in alveolar bone resorption)
*Streptococcus mutans*	---	Induces IL-1β production via inflammasome activation in THP1 macrophages [164]
	lipoteichoic acid purified	Induces TNF-α and nitric oxide (NO) production in RAW264.7 murine macrophages [165]
	Outer membrane vesicles (OMVs) purified	*S. mutans* OMVs increased production of IL-1β, IL-6, TNF-α and IL-8 (especially IL-1β) in THP1 cells; also reduces macrophage phagocytosis [166]
*Porphyromonas gingivalis*	Outer membrane vesicles (OMVs)	OMV-stimulated macrophages produce a large amount of TNF-α, IL-12p70, IL-6, IL-10, IFNβ, and nitric oxide; OMVs can also induce NK-KB activation 9 [167]
	---	Activates NLRP3 and AIM2 inflammasomes in THP1 cells via TLR2 and TLR4 signaling, leading to IL-1β secretion and pyroptotic cell death (pro-inflammatory cell death; apoptosis = non-inflammatory) via caspase-1 activation [168]
	Outer membrane vesicles (OMVs) purified	Macrophages require a second signal (e.g., exogenous ATP), whereas monocytes (and THP-1 cells) only require one signal for inflammasome activation. This distinction may neatly explain why *P. gingivalis* activation of the inflammasome and IL-1β production has been demonstrated in THP-1 and Mono-Mac-6 cell lines (Bostanci et al., 2009; Hamedi et al., 2009), as well as in human monocytes (Huang et al., 2009; Jung et al., 2015) [169]. In comparison, studies in mature macrophage populations found that *P. gingivalis* fails to activate the inflammasome (Taxman et al., 2012; Slocum et al., 2014) unless stimulated with a secondary signal. Used murine bone-marrow-der. macrophage (BMM); human monocyte-der. macrophage (MDM) - *P. gingivalis*-infected macrophages only weakly activated cytokine production and did not activate the inflammasome or induce pyroptosis. - *P. gingivalis* and its OMVs induce a metabolic shift in macrophages from oxidative phosphorylation to glycolysis. - *P. gingivalis* OMVs activate pro-inflammatory cytokine production (IL-6, IL-12p70, TNF-a, IFN-β [and NO]), inflammasome signaling, and pyroptotic cell death in macrophages.
*Fusobacterium nucleatum*	---	*Fn*-challenged Mφ (unpolarized mac, THP1) had significantly lower mRNA level of M1 markers iNOS and TNF-α but significantly higher mRNA level of M2 markers IL-10 and CD206 = suggests *Fn* may be involved in M2-like polarization [170]
	Outer membrane vesicles (OMVs) purified	Secreted OMVs can activate TLR4 and downstream targets ERK, CREB, and NF-κB, which promotes pro-inflammatory cytokine production. These effects were observed in colonic HT29 cells, as well as in human colonoid (organoid) monolayers [129].
	---	Activates both TLR2 and TLR4 in bone-marrow-derived macrophages to stimulate IL-6 production (and TNF-α) [171]
	Fn-cell wall; Lipopolysaccharides	- Gingival epithelial cells’ gene expression studied after stimulation with commensal FnCW. Up-regulated genes of defense included chemokines IL-8, CXCL1, CXCL3, CXCL5, and CXCL10 - Lipopolysaccharides from *F. nucleatum* can drive the production of inflammatory cytokines, such as IL-1α, IL-1β, IL-6, IL-8, and MMPs, through the activation of the translocation of the NF-κB gene into the nucleus [172]
	Two more promising papers: - “Fusobacterium nucleatum Contributes to the Carcinogenesis of Colorectal Cancer by Inducing Inflammation and Suppressing Host Immunity” [173] - “Withaferin A inhibits inflammatory responses induced by Fusobacterium nucleatum and Aggregatibacter actinomycetemcomitans in macrophages [174]	
*Streptococcus salivarius K12*	Salivaricin A2 and B (lantibiotics = antimicrobial peptides)	Salivaricins inhibit other pathogens; K12 inhibits NF-KB pathway/inflammation and stimulates type I and II interferon responses (type 2 = INF-Gamma!) “Our analyses…indicated that K12 didn’t initiate synthesis of proinflammatory cytokines/chemokines, nor did it regulate genes involved in responses to such molecules.” [131]
*Lactobacillus reuteri*	Membrane vesicles	MVs inhibit LPS-induced macrophage polarization towards pro-inflammatory phenotype, promote their polarization towards anti-inflammatory phenotype, and reduce inflammation levels in vitro 9 [175]
		“*L. reuteri* and inactivated *L. reuteri* treatment enhance macrophage phagocytic activity to kill intracellular pathogens (Figure 3A,B). Previous studies have also confirmed that *Lactobacillus* can activate macrophages, enhance phagocytosis ability and inhibit intracellular pathogen survival, thus playing a role in resisting pathogens [24,25].”
*Streptococcus oralis*	Hydrogen peroxide (H_2_O_2_)	Cytotoxic, kills monocytes and epithelial cells [176]

## 7. Future Directions for Therapeutic Innovation

Oral wound healing represents a highly coordinated process involving immune signaling, microbial ecology and tissue regeneration [177]. Traditionally research efforts have emphasized the role of growth factors, scaffolds and re-epithelization mechanisms. However, emerging studies now highlight the critical importance of the immune–microbiome axis, particularly the plasticity of macrophages and the influence of microbial communities on inflammation and healing outcomes.

A central finding from both experimental and clinical studies is the necessity for timely macrophage polarization from a pro-inflammatory M1 phenotype to an anti-inflammatory, tissue-repairing M2 phenotype [10]. Successful transition between these macrophage states is vital for resolving the initial inflammatory response and facilitating the proliferative and remodeling phases essential for tissue integrity. Dysregulation of this polarization, often triggered by persistent microbial imbalance or chronic pro-inflammatory signaling, is a key mechanism underlying delayed healing in oral pathologies such as periodontitis [178] and diabetic ulcers [179].

During the inflammatory phase of wound healing, M1 macrophages, characterized by surface markers such as CD86, MHC class II and CCR7, release pro-inflammatory cytokines IL-1β, IL-6, TNF-α and inducible nitric oxide (iNOS) to eliminate pathogens and recruit immune cells for debris clearance. However sustained M1 activation contributes to chronic inflammation and delayed healing [12]. In contrast, during the proliferative phase, polarization towards M2 macrophages, marked by CD163, CD206, PD-L1 and PD-L2, supports tissue repair, angiogenesis, fibroblast activation, and extracellular matrix deposition, particularly important for minimizing scarring and promoting vascularization [180]. In the remodeling phase, M2 macrophages contribute to collagen deposition and tissue homeostasis [181]. Impaired or dysregulated macrophage activity, therefore, represents a significant barrier to efficient wound healing.

Recent studies have demonstrated that the oral microbiome is a key player in oral wound repair [21]. In a state of eubiosis, balanced microbial communities support immune homeostasis, reduce inflammation, and inhibit pathogenic overgrowth, thereby promoting efficient healing [158]. In contrast, dysbiosis, characterized by microbial imbalance, leads to persistent inflammation, increased susceptibility to infection, and impaired tissue repair [182]. Probiotic interventions aimed at restoring microbial balance have demonstrated potential in enhancing angiogenesis, promoting structural integrity, and supporting tissue regeneration [81].

Beneficial microbes such as *Lactobacillus reuteri* and *Bifidobacterium breve* have been shown to promote M2 macrophage polarization, reduce oxidative stress and increase the expression of anti-inflammatory cytokines like IL-10 and TGF-β [124]. These immunomodulatory effects are mediated through microbial metabolites, outer membrane vesicles and peptides that engage host pattern recognition receptors (PRRs) [183]. Nevertheless, not all probiotic therapies yield consistent clinical outcomes. For instance, while some clinical trials report modest reductions in postoperative inflammation following probiotic lozenge use, improvements in healing kinetics have been limited [156]. These findings highlight the complexity of host–microbe interactions and the need for optimized delivery strategies tailored to the wound environment.

Conversely, pathogenic bacteria such as Porphyromonas gingivalis and Fusobacterium nucleatum exacerbate wound pathology by sustaining M1-type inflammation through TLR-dependent pathways and activation of inflammation [184]. These pathogens effectively disrupt wound homeostasis, prolonging the inflammatory phase, and hinder progression to repair stages.

**Figure 4:**
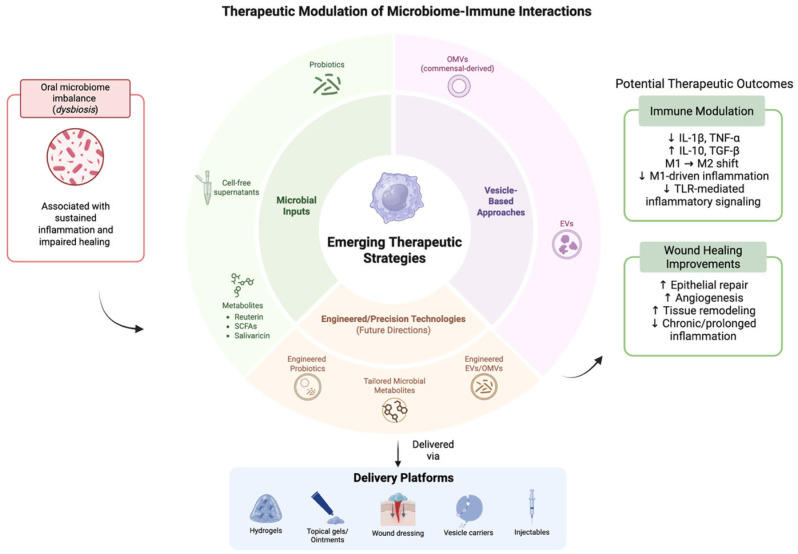
Emerging therapeutic approaches to modulate microbiome–immune crosstalk. The above figure was created with BioRender.com. Goudy, S. (2026) https://BioRender.com/gfoio8n.

By combining metagenomic, metatranscriptomic, proteomic and metabolomic profiling of microbial communities with host single-cell and spatial transcriptomics/omics, researchers can simultaneously characterize which microbial taxa are present, what genes and metabolites they express, and how host immune cells respond at high resolution [185]. Recognizing the tissue architecture while examining both host and microbial molecular states enables the linkage of microbial signals to define the phenotypes of immune cells within their native contexts, thus going beyond traditional single-cell profiling techniques that disrupt samples through spatial multi-omics. Single-cell RNA sequencing of oral mucosa revealed macrophage dynamics in clearing of microbes by macrophage subpopulations in the inflammatory and remodeling phase [186]. On the computational side, sophisticated AI and machine learning models tailored for high-dimensional multi-omics data can combine these diverse datasets (microbial genomics/transcriptomics/metabolomics + host transcriptomics/epigenomics + spatial context) to deduce networks of interactions between microbes and hosts, pinpoint crucial metabolites or signals derived from microbes that are linked to immune activation or suppression, and differentiate individual variations that might affect disease susceptibility or healing responses [187]. Combining multi-omics, spatial or single-cell sequencing and AI-powered integrative analytics creates a robust, detailed approach to unravel the mechanisms of microbe–immune interactions [188]. This holds the potential to reveal new strategies for microbiome-driven modulation of macrophage activity and enhanced oral health treatments (Figure 4).

Macrophages are highly responsive to local environmental cues, including cytokines and microbial signals, which shape their functional phenotypes during wound healing. These observations underscore the potential of microbiome-targeted immunomodulatory strategies as promising therapeutic approaches for managing chronic and impaired oral wounds.

Bringing scientific insights into clinical therapies is further challenged by the ever-changing oral environment, where saliva, chewing, and microbial competition reduce how well therapeutic agents stay in place. This has led to a focus on new delivery systems, including mucoadhesive hydrogels, nanofiber scaffolds, nanoparticles, and smart biomaterials, that can provide sustained, targeted release of immune modulators at the fistula site. New approaches such as 3D bioprinting and tissue-engineered constructs with built-in immune cues also offer promising solutions for repairing complex defects and achieving long-term tissue integration. In the end, combining multi-omics analysis, advanced biomaterials, and targeted immune therapies could pave the way for personalized, biologically informed treatments that lower recurrence rates and improve outcomes for people with oronasal fistula.

Further investigations are warranted to define the specific microbial components and host signaling pathways that optimize macrophage function and promote regenerative healing. Future therapies may involve precision probiotic formulations, microbial-derived metabolites or engineered extracellular vesicles to specifically modulate immune response in chronic wound conditions.

This schematic illustrates emerging microbiome-informed therapeutic approaches, including microbial inputs, vesicle-based strategies, and engineered modalities, along with their delivery platforms. These interventions aim to modulate immune responses and support wound healing processes in the oral environment. Potential therapeutic outcomes shown are based on preliminary experimental findings. Their efficacy remains variable and is influenced by complex host–microbiome–immune interactions.

## 8. Conclusions

Advancing targeted therapies for oral wound healing requires a comprehensive understanding of the dynamic crosstalk between the oral microbiome, host immune responses, and tissue regeneration pathways. This review highlights key mechanistic insights demonstrating how microbial composition and microbe-derived metabolites regulate inflammatory resolution, macrophage polarization, epithelial barrier restoration, and angiogenesis, all critical determinants of effective wound repair. While emerging preclinical evidence supports microbiome-based and immune-targeted therapeutic strategies, clinical translation remains limited by wound heterogeneity, inter-individual microbiome variability, and the absence of standardized regulatory and clinical trial frameworks. Future research should prioritize inclusion of underexplored microbial components such as the oral virome and mycobiome, development of personalized and precision probiotic formulations, and well-designed longitudinal clinical trials to establish efficacy, safety, and dosing strategies. Integrating advances in microbiology, immunology, biomaterials, and systems biology will be essential to overcome current gaps and unlock the full therapeutic potential of microbiome-based interventions for oral wound healing.

## Data Availability

No new data were created or analyzed in this study. Data sharing is not applicable to this article.

## Abbreviations

The following abbreviations are used in this manuscript:

AI	Artificial Intelligence
AIM2	Absent in Melanoma 2
ATP	Adenosine Triphosphate
BCN	Bacterial Cellulose Nanocomposite
BMM	Bone Marrow-Derived Macrophages
BV	Bacterial Vaginosis
CAR-T	Chimeric Antigen Receptor T cells
CCR7	C-C Chemokine Receptor Type 7
CD	Cluster of Differentiation
CF	Cystic Fibrosis
CX3CR1	C-X3-C Motif Chemokine Receptor 1
CXCL	C-X-C Motif Chemokine Ligand
ECM	Extracellular Matrix
EGF	Epidermal Growth Factor
FGF	Fibroblast Growth Factor
GM-CSF	Granulocyte–Macrophage Colony-Stimulating Factor
HAEPS	Hyaluronic Acid-Encapsulated Probiotic System
HIF-1α	Hypoxia-Inducible Factor-1 Alpha
IBS	Irritable Bowel Syndrome
IFN-β	Interferon Beta
IFN-γ	Interferon Gamma
IGF-1	Insulin-Like Growth Factor-1
IL	Interleukin
IL-1RA	Interleukin-1 Receptor Antagonist
iNOS	Inducible Nitric Oxide Synthase
LPS	Lipopolysaccharide
MHC	Major Histocompatibility Complex
MDM	Monocyte-Derived Macrophages
MMP	Matrix Metalloproteinase
MVs	Membrane Vesicles
NETs	Neutrophil Extracellular Traps
NF-κB	Nuclear Factor Kappa-Light-Chain-Enhancer of Activated B Cells
NGF	Nerve Growth Factor
NO	Nitric Oxide
OMVs	Outer Membrane Vesicles
ONF	Oro-Nasal Fistula
OSCC	Oral Squamous Cell Carcinoma
PAMPs	Pathogen-Associated Molecular Patterns
PD-L1	Programmed Death-Ligand 1
PD-L2	Programmed Death-Ligand 2
PDGF	Platelet-Derived Growth Factor
PEG	Polyethylene Glycol
PG	Peptidoglycan
PRRs	Pattern Recognition Receptors
ROS	Reactive Oxygen Species
SCFAs	Short-Chain Fatty Acids
SNPs	Single-Nucleotide Polymorphisms
STAT1	Signal Transducer and Activator of Transcription 1
TAMs	Tumor-Associated Macrophages
TGF-α	Transforming Growth Factor Alpha
TGF-β	Transforming Growth Factor Beta
Th17	T Helper 17 Cells
TIMPs	Tissue Inhibitors of Metalloproteinases
TLR	Toll-Like Receptor
TNF-α	Tumor Necrosis Factor Alpha
VEGF	Vascular Endothelial Growth Factor
